# Effect of hyperglycemia treatment on complications rate after pediatric cardiac surgery

**DOI:** 10.34172/jcvtr.2022.05

**Published:** 2022-03-12

**Authors:** Bahman Naghipour, Mahdieh Bagerpour, Kamran Shadvar, Samad E.J. Golzari, Gholamreza Faridaalaee

**Affiliations:** ^1^Department of Anesthesiology and Intensive Care, Medical Faculty, Tabriz University of Medical Sciences, Tabriz, Iran; ^2^Department of Gynecology and obestetric, Tabriz University of Medical Sciences, Tabriz, Iran; ^3^Emergency Medicine Research Team, Faculty of Medicine, Tabriz University of Medical Sciences, Tabriz, Iran; ^4^Department of Emergency Medicine, Maragheh University of Medical Sciences, Maragheh, Iran

**Keywords:** Hyperglycemia, Pediatrics, Heart, Thoracic Surgery, Cardiac Surgical Procedures, Treatment and Complications

## Abstract

***Introduction:*** The goal of this study was to elucidate harmful complications of intraoperative hyperglycemia following children cardiac surgery and benefits of insulin administration for accurate blood sugar controlling.

***Methods:*** this study is a Randomized clinical trial. We conducted this study in the operating room of shahid madani hospital. Fifty patients who were children under 12 years old undergone cardiac surgery using cardiopulmonary bypass (CPB). Intraoperative insulin infusion was administered intravenously targeting blood sugar levels of 110-140 mg/dL. Blood sugar and arterial blood gas (ABG) were measured every 30 min during operation.

***Results:*** Inotropes were used less in the study than the placebo group during surgery. The means of hospitalization and extubation time were more in the placebo group than the study group(*P* =0.03) and (*P* =0.005), respectively. However, the mean time of hospitalization in the ICU ward did not differ significantly between the two groups.

***Conclusion:*** Hyperglycemia has a relation with long time of intubation and hospitalization in ICU. These findings suggest the positive effect of accurate blood sugar control on reducing complication and hospitalization time in children undergoing cardiac surgery.

## Introduction

Congenital heart defects are one of the common birth defects^1^ and most affected children require cardiac surgery. Morbidity and mortality following operation of infants and young children are partly high and these patients require different intra-operative management than their adult counterparts.^2^ Diagnosis and management of amendable risk factors throughout the surgeries are an important step which contributes to proper postoperative outcomes.^1^ Hyperglycemia is a state that may occur after cardiac surgery in this group of children.^3-5^ Hyperglycemia has been reported to affect up to 90% of the patients in some studies.^4,6-8^ The incidence of hyperglycemia is partially due to the increase in glucose production by liver, excretion of counter-regulatory hormones and peripheral resistance to insulin.^9-11^

Several studies for evaluating frequency and symptoms of hyperglycemia demonstrate a correlation between hyperglycemia and morbidity and mortality rates.^3,12-14^ In contrast, some studies have not demonstrated a distinct association between hyperglycemia and increase in mortality or major complications.^3^ Several protocols have been proposed for controlling blood sugar in children with critical illness;^13,15,16^ however, some questions still exist about the optimum range for blood sugar control and dangers of hypoglycemia originating it. Several studies have reported improvement in the accurate blood sugar control with insulin. The use of accurate blood sugar control in pediatric ICU due to rise of hypoglycemia is not very common.^17^ There is a lack of consensus on intra-operative hyperglycemia, harmful complications following children cardiac surgery and insulin administration for accurate blood sugar controlling.^7,14,18,19^

## Materials and methods

### Design

This study is a randomized clinical trial.

### Participant

Inclusion criteria were children under 12 years old undergoing cardiac surgery using cardiopulmonary bypass (CPB). Exclusion criteria were diabetic patients, lack of informed consent by surrogates, emergency surgeries, insufficiency of other organs (lung, kidney or liver), and patients with ejection fraction (EF) of less than 40%. 50 patients were divided into two groups of the placebo and study.

### Intervention

Dexamethasone was administered intravenously 0.1mg/kg to reduce inflammatory response to the pump. Serum (Dextrose 5%/Nacl 0.45) was administered using an infusion pump 2 mL/kg/hour for all children. Anesthesia was induced using midazolam 0.1 mg/kg, fentanyl 5 µg/kg, Cis-atracurium 0.2 mg/kg, and lidocaine 1 mg/kg; anesthesia was maintained using Total Intravenous Anesthesia (TIVA) which consisted of midazolam 1 µg/kg/min, fentanyl 2 µg/kg/hour, and Cis-atracurium 0.2 mg/kg/hour. All children were monitored for pulse oximetry, ECG, Invasive Blood Pressure, Central Venous pressure (CVP), and End Tidal CO_2_ (ET CO_2_). A 50mL syringe of normal saline containing 0.1 U/mL insulin was administered intravenously and infused targeting at blood sugar levels of 110-140mg/dL using the protocol presented in Table 1. The infusion was continued until the end of the operation and was held while transferring to the ICU. For the placebo group, there was no accurate blood sugar control by insulin infusion. To consider ethical issues, blood sugar was controlled by regular insulin bolus doses based on a routine insulin protocol in case of rise in the blood sugar to more than 200 mg/dL. Dextrose 5% serum and hypertonic glucose (50%) were prepared for hypoglycemia incidence. Blood sugar and ABG were measured every 30 min during operation. Fasting Blood Sugar (FBS) more than 126 mg/dL was considered as hyperglycemia. Blood sugar decline was considered as less than 60 mg/dL in each calculation period.

**Table 1:** Protocol of blood glucose control

**Blood sugar (mg/dL)**	**Insulin solution infusion speed (mL/h)**	**Other administration**
110-140	0.5	-
140-150	1	-
150-160	2	-
160-180	3	-
180-200	4	-
> 200	5	-
80-110	Insulin interrupt	-
60-80	Insulin interrupt	Infusion of dextrose 5%
< 60	Insulin interrupt	Infusion of hypertonic glucose 50% 0.2 mL/kg

### Randomization

Patients’ randomization was performed by online software (random.org). Anesthesiologists and nurses were not aware of children groups in the ICU.

### Outcome

Blood sugar was assessed every one hour until four hours and then every four hours during hospitalization in the ICU. Demographic information, hemodynamic condition, serum blood sugar during surgery, ventilation condition after surgery, incidence of possible symptoms after surgery, and death were registered in data collection form.

### Statistical analysis

Data were analyzed using SPSS 16. Descriptive statistical methods were used for statistical analyses. Comparison between qualitative findings was performed using Chi square test and in case of need to accurate method, Fischer’s exact test was used. The quantitative data were analyzed by an independent *t*-test. Lactate and glucose concentration levels during follow up periods were evaluated by repeated measure of ANOVA. *P* value less than 0.05 was considered statistically significant.

## Results

### Participants

**Figure 1:**
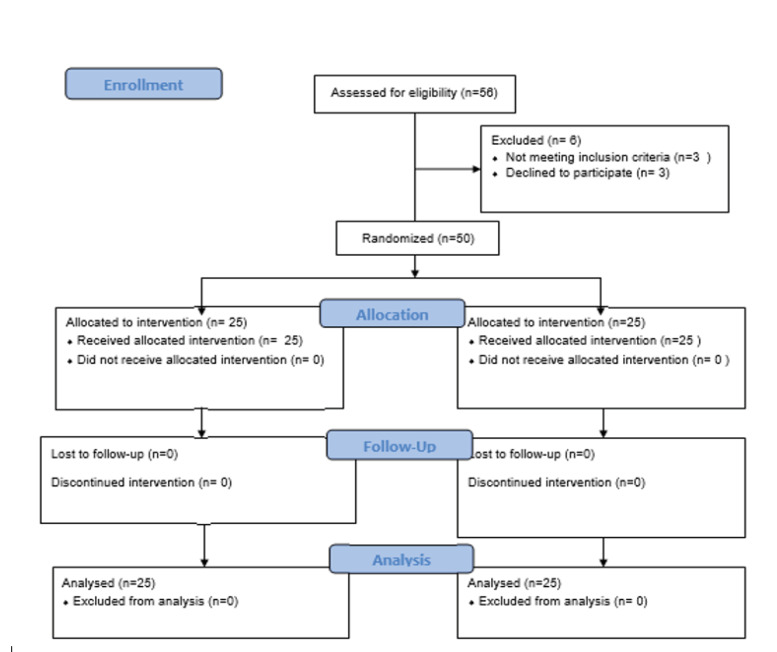
Consort flow diagram

The flow diagram of participant is shown in Figure 1. Finley 50 patients enrolled in this study.

### Basic data

Demographic and basic data of the patients are demonstrated in Table 2; there was no significant difference between two groups in this regard. Prostaglandins were not used in children. Steroid before surgery was administered in 9 children (36%) of the placebo and 10 children (40%) of the study groups; there was no significant difference between two groups. Mean of surgery time was 336.00 ± 68.19 min and 313.20 ± 54.82 min the placebo and study groups, respectively; there was no significant difference between two groups. Types of the surgeries are demonstrated in Table 3.

**Table 2.**

**Table 2:** Demographic data of patients

**Findings**		**Placebo**	**Study**	***P*** ** value**
Age (Mean ± SD)		3.32 ± 2.57	3.08 ± 2.83	0.74
Gender N (%)	Male	15 (60%)	14 (56%)	0.77
	Female	10 (40%)	11 (44%)	
Weight(kg)(Mean ± SD)		12.70 ± 4.77	11.66 ± 5.51	0.47
Height(cm) (Mean ± SD)		88.28 ± 25.81	88.80 ± 21.16	0.93

**Table 3:** Type of surgery in two groups

**Type of surgery**	**Placebo**	**Study**	**Total**
VSD closure	5 (20%)	6 9((24%)	11 (22%)
ASD closure	4 (16%)	5 (20%)	9 (18%)
VSD & PS Restoration with RV myotomy	9 (36%)	7 (28%)	16 (32%)
PDA & ASD & PDA closure	0	3 (12%)	3 (6%)
Glen shunt	1 (4%)	0	1 (2%)
PS repair	1 (4%)	2 (8%)	3 (6%)
Full repair	3 (12%)	0	3 (6%)
Glen Shunt single V	2 (8%)	0	2 (4%)
Valvectomy	0	2 (8%)	2 (4%)

Abbreviations: ASD, atrial septal defect; PS, pulmonary stenosis; RV, right ventricle; PDA, patent ductus arteriosus; VSD, ventricle septal defect

### Outcome

Mean of cardiopulmonary bypass (CPB) time was 110.80 ± 44.50 min and 94.24 ± 46.76 min in the placebo and study groups, respectively that there was no statistically significant difference. Aortic clamp was used in 23 and 24 children of the placebo and study groups, respectively. Mean of clamping time was 81.47 ± 38.58 min and 63.79 ± 34.98 min in the placebo and study groups, respectively that there was no statistically significant difference. Deep hypothermia and circulatory arrest were performed in 2 (8%) patients of the placebo group. Vasopressors (phenylephrine) were required during pumping in 11 (44%) and 4 children (16%) of the placebo and study groups, respectively; there was statistically significant difference between two groups (*P* = 0.03). Sternum was closed in all except 3 children from the placebo group. Steroid was administered in the 22 (88%) and 24 children (96%) of the placebo and study groups, respectively, and that there was no statistically significant difference.

Hypoglycemia was observed in 4 (16%) and 5 children (20%) of the placebo and study groups, respectively, and that there was no statistically significant difference between two groups. Blood sugar increased in the study group until 6 hours after ICU transfer; however, it later decreased. Blood sugar increased in the placebo group after induction and started to decline from the second day in the ICU; there was a statistically significant difference between two groups (*P* = 0.04). Serum lactate concentration increased until the end of pump in the study group which was less compared to the placebo group; however, serum lactate concentrations declined later. There was statistically significant difference between two groups regarding lactate concentrations after induction (*P* = 0.04), after pump (*P* = 0.003), and 6 hours after ICU transfer (*P* = 0.003).

The maximum administered dose of milrinone was 0.43 ± 0.40 µg/kg/min and 0.27 ± 0.11 µg/kg/min in placebo and study groups, respectively. There was no significant difference. The maximum administered dose of dopamine was 9.85 ± 3.63 µg/kg/min and 5.753.16 µg/kg/min in the placebo and study groups, respectively. Dopamine was used more in the placebo than the study group (*P* = 0.003). Inotropes were used less in the study than the placebo group during surgery (*P* = 0.005). In ICU, inotropes were used less in the study than the placebo group (*P* = 0.02).

The mean creatinine level in the placebo group (0.71 ± 0.17 mg/dL) was more than the study group (0.55 ± 0.15 mg/dL) on the second day (*P* = 0.006). AST and ACT levels are demonstrated in Table 4. Mean of AST increase from day 1 to 2 was less in the study than the placebo group (*P* = 0.01). Mean of AST increase from day 1 to 3 was less in the study than the placebo group (*P* = 0.009). the mean time of hospitalization in the ICU was 5.31 ± 3.84 days and 4.36 ± 3.75 days in the placebo and study groups, respectively. There was no statistically difference. The mean time of hospitalization was more in the placebo than the study group; there was statistical difference between two groups (*P* = 0.03).

**Table 4:** AST and ACT level

	**Day**	**Placebo**	**Study**	***P*** ** value**
AST	1	17.77 ± 4.38	15.54 ± 2.12	0.03
AST	2	25.38 ± 7.92	18.83 ± 2.85	0.001
AST	3	28.60 ± 12.75	19.66 ± 2.29	0.003
ACT	1	127.70 ± 9.56	131.08 ± 5.19	0.12
ACT	2	126.00 ± 5.51	125.37 ± 4.94	0.69
ACT	3	120.50 ± 6.33	118.66 ± 5.93	0.32

Abbreviations: AST, aspartate aminotransferase, a liver function test; ACT, activated clotting time test

The mean of extubation time was 41.05 ± 30.27 hours and 19.94 ± 18.12 hours in the placebo and study groups, respectively; there was statistically difference between two groups (*P* = 0.005). Temporary pacing after operation was required in 11 (44%) and 3 (12%) children from the placebo and study groups, respectively (*P* = 0.01); there was statistically significant difference between two groups. Reoperation was required in 5 (20%) and no children from the placebo and study groups, respectively (*P* = 0.01); there was a statistically significant difference between two groups. Complication rates after the operation are demonstrated in Table 5.

**Table 5:** complication rate after the operation

**Symptoms**	**Placebo**	**Study**	***P*** ** value**
Overall	8 (32%)	2 (8%)	0.03
Renal failure	1 (4%)	1 (4%)	-
Stroke	1 (4%)	0	0.9
Epilepsy	0	0	-
Postoperative infection	4 (16%)	1 (4%)	0.34
Cardiopulmonary arrest	3 (12%)	1 (4%)	0.6
Death	4 (16%)	1 (4%)	0.34

## Discussion

Children undergoing repair surgery for restoration of the congenital heart disease are at risk of hyperglycemia. Despite being a controversial issue, diagnosis and management of modifiable risk factors result in proper postoperative outcomes in both children and adults. Using accurate blood sugar control in ICU hospitalized children is not common due to the increased risk of hypoglycemia in these children.^17^

In this study, Inotropes were used less in the study than the placebo group during surgery. The means of hospitalization and extubation time were more in the placebo group than the study group (*P* = 0.03) and (*P* = 0.005), respectively. Also, hyperglycemia and hypoglycemia frequency were 56% and 16% in the placebo group, respectively. Hypoglycemia frequency was 20% in the study group. There was no statistical difference between the two groups. Verhoeven et al reported hyperglycemia in 52% of children after surgery.^5^ Accordingly, Moga et al demonstrated hyperglycemia in 90% of their study patients; however, hyperglycemia diminished without insulin administration 72 hours after surgery.^4^ Falcao et al illustrated slight and severe hyperglycemia in patients (97% and 78%).^12^ In Preissig et al study, hyperglycemia prevalence was 84%.^13^ Hyperglycemia prevalence was lower in our study than mentioned studies in which the patients were either given insulin or not. Administration of corticosteroids before and after surgery and different stresses might contribute to this increased hyperglycemia prevalence in the similar studies.

Various studies have correlated hyperglycemia with death in critical patients.^20-24^ Vlasselaer et al suggested that accurate blood sugar control before and during operation is protective and decreases inflammatory responses.^25^ In contrast, Agus et al reported no significant difference regarding complications with or without treatment with insulin;^1^ in another study, it was demonstrated that accurate blood sugar control during cardiac surgery can reduce infection risk in patients older than 60 days of age.^26^ Yates et al reported that hyperglycemia duration has relation with long hospitalization time in ICU and hospital.^14^ These findings suggest the positive effect of accurate blood sugar control on reducing complication and hospitalization time in children undergoing cardiac surgery. Like our result in this study, Rodolfo J. Galindo et al showed that glycemic control in patients with diabetes reduces perioperative complications during cardiac surgery. Also Camila Perez de Souza Arthur et al showed the same result in diabetic patients.^27,28^ However, our study was in non-diabetic patients and showed in non-diabetic patients like diabetic patient’s accurate glycemic control reduces perioperative complications.

## Conclusion

Hyperglycemia has a relation with long time of intubation and hospitalization in ICU. These findings suggest the positive effect of accurate blood sugar control on reducing complication and hospitalization time in children undergoing cardiac surgery.

Limitation: the low sample size was our study limitation.

## Acknowledgements

All authors acknowledge the Tabriz University of Medical Sciences for suporting this study and the operating room staff of shahid madani hospital for cooperation in this study.

## Funding

This research did not receive any specific grant from funding agencies in the public, commercial, or not-for-profit sectors.

## Ethical approval

This study was approved by ethic committee of Tabriz University of medical Sciences (92139). The study was registered in Iran RCT center (IRCT2014052316117N2). Informed consent was obtained from all parents of patients.

## Competing interests

The authors declare that they have no competing interests.
